# Supplementary material for: Isolated Severe Neutropenia in Adults, Evaluation of Underlying Causes and Outcomes, Real-World Data Collected over a 5-Year Period in a Tertiary Referral Hospital
Source: Medicina (Kaunas). 2024 Sep 26;60(10):1576. doi: 10.3390/medicina60101576 (PMC11509619; doi:10.3390/medicina60101576)
Supplement: Supplementary file 1 [file medicina-60-01576-s001.zip › medicina-3170944-supplementary.pdf]

Supplementary data

Supplementary Table S1: Diagnostic investigations and findings

**Diagnostic tool**	*n* *	**Etiology of neutropenia**	**Results**
**Bone marrow testing**	20 (28%)	AIN, *n*=4<br>CIN, *n*=11<br>SCN, *n* =4<br>DIN, *n* =1	**well-differentiated myelopoesis:** n=12/20 (60%)<br>**left-shifted myelopoesis**: n=8/20 (40%)<br>**myeloid dysplasia**: n=1 (5%)<br>**increased blasts:** n=0 (0%)<br>**normocellular:** n=15/20 (75%<br>**hypercellular:** n=5/20 (25%)
**Cytogenetics**	9(12.8%)	AIN, *n* =3<br>CIN, *n* =4<br>SCN, *n* = 2	All normal karyotype
**Spleen ultrasound**	7 (10%)	AIN, *n* =2<br>CIN, *n* =2<br>DIN, *n* =2<br>Post-infectious neutropenia, *n* =1	median spleen size 14.4cm.<br>7 patients with mild splenomegaly.
**Antineutrophil-antibodies**	3 (4.3%)	AIN, *n* =2<br>CIN, *n* =1	2 positive, 1 negative
**Molecular investigations**<br>Hereditary Neutropenia Panel	5 (7.1%)	SCN, *n* =4<br>CIN, *n* =1	see table

Somatic mutations myeloid neoplasms NGS Panel	4 (5.7%)	SCN, *n* =1	DNMT3A Mutation, VAF 4%
		CIN, *n* =3	Negative
**Flow cytometry from peripheral blood**<br>Lymphocyte screening	23 (32%)	AIN, *n* =6	14/23: normal<br>9/23: quantitative T- and B-Cell subset abnormalities
		CIN, *n* =7	
		SCN, *n* =3	
		DIN, *n* =6	
		Post-infectious neutropenia, *n* =1	
PNH clone screen	2 (2.8%)	CIN, *n* =2	absence of PNH clones
Monocyte-subsets	2 (2.8%)	CIN, *n* =1<br>DIN, *n* =1	Normal
**Flow cytometry from**<br>**Bone marrow**	9(12.8%)		No evidence for lymphoid neoplasms
Lymphocyte screening	6 (8.5%)	AIN, *n* =2<br>CIN, *n* = 2<br>SCN, *n* =1<br>DIN, *n* =1	
Blasts screen	7 (10%)	AIN, *n* =2<br>CIN, *n* =3<br>DIN, *n* =2	Normal/ no evidence of increased blasts
Both lymphocyte and blasts screening	5 (7.1%)		

Abbreviations:*Number of patients who underwent testing (% of whole cohort), AIN, autoimmune neutropenia; CIN, chronic idiopathic neutropenia; SCN, severe congenital neutropenia; DIN, drug induced neutropenia.

Overall, 20 of 70 (28%) patients underwent bone marrow examination. 19 of these had chronic neutropenia (lasting >3 months). Molecular genetic analysis was performed in 8 of the 70 patients in our cohort, these were all patients with chronic neutropenia.

Inherited neutropenia gene sequencing panel was carried out in 5 patients. In our laboratory, the genes tested are: AK2, CSF3R, CXCR4, ELANE/ELA2, GATA2, GFI1, G6PC3, HAX1, JAGN1, LAMTOR2/MAPBPIP (p14), SAMD9, SAMD9L, SRP54, SRP72, VPS45 and WAS.

Mutations associated with congenital neutropenia were detected in 4 patients. Next-generation sequencing (NGS) myeloid panel tests were conducted in 4 patients. Only one test detected a DNMT3A mutation with a low allele-burden in a patient with SCN.

Supplementary Table S2: Detailed analysis of patients with metamizol induced agranulocytosis

Patient	Age at diagnosis	Sex	Duration of neutropenia <0.5 G/l in days	G-CSF	Antibiotic therapy	Duration of hospitalisation in days
**1**	51	male	8	no	Cefepime	6
**2**	21	female	6	yes	Cefepime Metronidazole	11
**3**	34	male	2	no	Cefepime Metronidazole	4
**4**	42	female	3	yes	Co Amoxicillin	2
**5**	36	male	3	no	Azithromycin	4
**6**	25	female	4	no	Ceftriaxon	4
**7**	18	male	2	no	Cefepime	5
**8**	29	female	1	no	none	5
**9**	21	female	2	no	Cefepime	5
**10**	64	male	2	no	Cefepime Metronidazole	4
**11**	23	female	4	yes	Cefepime	5
**12**	20	female	3	no	Cefepime	5
**13**	55	Male	2	yes	Cefepime	3
**14**	31	female	2	no	none	0
